# Association between diet-related knowledge, attitudes, behaviors, and self-rated health in Chinese adult residents: a population-based study

**DOI:** 10.1186/s12889-020-08896-y

**Published:** 2020-05-19

**Authors:** Ying Yang, Di He, Liuyi Wei, Shizhen Wang, Lei Chen, Mi Luo, Zongfu Mao

**Affiliations:** 1 School of Health Sciences, Wuhan University, 115# Donghu Road, Wuhan, 430071 China; 2 Global Health Institute, Wuhan University, 115# Donghu Road, Wuhan, 430071 China; 3 Department of Nursing, Tongji Hospital, Tongji Medical College, Huazhong University of Science & Technology, 1095# Jiefang Avenue, Wuhan, 430030 China

**Keywords:** Diet, Knowledge, Attitudes, Behaviors, Self-rated health, China

## Abstract

**Background:**

Diet-related knowledge, attitudes, and behaviors (KABs) are important for building healthier dietary patterns. We conducted this study to (a) investigate diet conditions of Chinese adult residents from the perspective of knowledge, attitudes, and behaviors, and (b) assess the association between diet-related KABs and self-rated health.

**Methods:**

We analyzed the 2015 China Health and Nutrition Survey (CHNS) data. Individuals aged 18 years and older were included as study subjects (*n* = 12,814), assessing their diet-related knowledge, attitudes, behaviors, and self-rated health. Comparison of diet-related KABs in urban and rural residents was conducted using chi-square test. Ordinal logistic regression analysis was adopted to examine the association between diet-related KABs and self-rated health.

**Results:**

The proportion of knowing about the Chinese Food Pagoda (CFP) or the Dietary Guidelines for Chinese Residents (DGCR) was 27.1%. 34.3% of the participants were assessed as having adequate dietary knowledge literacy. 24.3% reported a positive attitude towards healthy eating. 27.6 and 65.9% of the participants reported proactively looking for nutrition knowledge and preferring eating fruits & vegetables, respectively. Chi-square test indicated that rural people experienced poorer diet-related knowledge, attitudes, and behaviors than urban residents (all *p*-values < 0.01). Regression analysis revealed that participants who knew about CFP/DGCR (*OR* = 1.11, 95% *CI* = 1.08–1.15), had adequate dietary knowledge literacy (*OR* = 1.12, 95% *CI* = 1.10–1.15), held positive attitude towards healthy eating (*OR* = 1.14, 95% *CI* = 1.09–1.19), proactively looked for nutrition knowledge (*OR* = 1.11, 95% *CI* = 1.08–1.15), and preferred eating fruits & vegetables (*OR* = 1.09, 95% *CI* = 1.07–1.12) had significantly better self-rated health.

**Conclusions:**

Chinese adult residents experienced poor diet-related knowledge, attitudes, and behaviors. Rural people had significantly worse diet conditions than urban residents. Better diet-related knowledge, attitudes, and behaviors were associated with higher self-rated health in Chinese adult residents.

## Background

Eleven million deaths (22% of all deaths among adults) and 255 million Disability Adjusted Life Years (DALYs) were attributable to dietary risk factors, based on the Global Burden of Disease Study 2017 [1]. Chinese people are confronting with the plight of the coexistence of under- and over-nutrition, and the incidence of nutrition-related diseases is high in China [2]. Chinese residents’ daily salt and edible oil intake and fat ratio were all higher than the recommended standards (WHO and Dietary Guidelines for Chinese Residents (DGCR)) in 2012 [2]. The rates of overweight and obesity showed an upward trend in Chinese adults, while 6.0 and 9.0% of Chinese adults still faced the problems of malnutrition and weight loss. To promote the solution of nutrition-related problems, Chinese government announced the implementation of “Healthy China Action (2019-2030)” in July 2019, in which “Appropriate Diet” was involved as one of fifteen major actions [3].

The theory of Knowledge, Attitude/Belief, and Behavior/Practice (KAB) was originally proposed to emphasize the vital role of knowledge, attitudes, and behaviors in health management [4]. KAB theory holds that individual health behavior is composed of three consecutive processes: acquiring knowledge, generating beliefs, and forming behaviors. KAB evaluation, which is considered the first step of behavioral education, can help health educators grasp individuals’ understanding, health beliefs, and actions taken regarding a specific health issue, and provide the scientific basis for the development of intervention plans. The knowledge of health-related behaviors was reported to influence individuals’ attitudes and practices in health management [5]. Specifically, assessing diet-related knowledge, attitudes, and behaviors was of vital importance in dietary health promotion at the population level [6–8], and thus it should be a necessary prerequisite for the implementation of dietary intervention in China.

Previous studies have revealed that diet-related behaviors can influence individuals’ health. For example, diet-related behaviors are associated with mortality and morbidity of non-communicable diseases [1], risk of certain diseases (cardiovascular disease [9, 10], metabolic syndrome [11–13], and cancer [9, 14]), and all-cause mortality [9]. Knowledge and attitudes indirectly affect individuals’ health status by promoting behavior change [15, 16]. However, no study has investigated the association between dietary behaviors and individual health in Chinese residents from the KAB theory perspective. For these considerations, we conducted this study to (a) investigate diet conditions of Chinese adult residents from the perspective of knowledge, attitudes, and behaviors, and (b) assess the association between diet-related knowledge, attitudes, behaviors and self-rated health.

## Methods

### Data source

The China Health and Nutrition Survey (CHNS) is a longitudinal survey and open cohort, which has been conducted since 1989 with multistage and random cluster procedures. The comprehensive dataset aims to explore the influences of nutrition, health, and family planning policies established by both national and local government agencies in China. CHNS investigates the impact of social and economic transitions in Chinese society on residents’ overall health and nutrition status. In the 2015 CHNS data, a total of 15,291 individuals were surveyed from nine provinces (Liaoning, Heilongjiang, Jiangsu, Shandong, Henan, Hubei, Hunan, Guangxi, and Guizhou) and three municipalities (Beijing, Chongqing, and Shanghai).

This study used the 2015 CHNS data. Individuals aged 18 years and older were included as study subjects (*n* = 12,814). After excluding those with missing socio-demographic characteristics, diet-related variables, and self-rated health information, a total of 12,814 subjects were involved in the final analysis.

This study used de-identified and publicly-available datasets from the official CHNS website (https://www.cpc.unc.edu/projects/china). Hence, approval from Institutional Review Boards was not required at the authors’ institution.

### Variables

#### Diet-related knowledge

Two indicators were applied to assess diet-related knowledge: knowing about the Chinese Food Pagoda (CFP) or the Dietary Guidelines for Chinese Residents (DGCR) and having adequate dietary knowledge literacy. The first indicator was calculated based on the question “Do you know about the Chinese Food Pagoda or the Dietary Guidelines for Chinese Residents? (yes/no)” The second indicator was computed from 17 dietary questions. Individuals with the actual dietary knowledge score ≥ 80% of the full score were defined as having adequate dietary knowledge literacy (i.e. the total score of the 17 dietary questions ≥14) [17, 18].

Seventeen dietary knowledge questions which coded as “strongly disagree”, “disagree”, “neutral”, “agree”, and “strongly agree” in the 2015 CHNS questionnaire were transferred into dichotomous variables. For seven negative items (Q2, Q4, Q6, Q12, Q14, Q15, Q16), the response of “strongly disagree” or “disagree” was scored 1 point, otherwise 0. For the other ten positive items, the response of “strongly agree” or “agree” was scored 1 point, otherwise 0. Cronbach’s alpha for the 17 dietary questions was 0.86 in this study.

#### Diet-related attitudes

The view on the importance of healthy eating was selected as the indicator of diet-related attitudes. Participants were asked: “How important is eating a healthy diet priority in your life: the most important, very important, neutral, not very important, or not important at all?” The response of “the most important” or “very important” represented positive attitude.

#### Diet-related behaviors

We considered two behaviors related to diet: looking for nutrition knowledge and eating fruits & vegetables. The behavior of looking for nutrition knowledge was measured by the question “Do you proactively look for nutrition knowledge (yes/no)?” The response of “yes” represented positive behavior. The behavior of eating fruits & vegetables was investigated by the question “How much do you like fruits & vegetables: like very much, like, neutral, dislike, or dislike very much?” The response of “like” or “like very much” represented positive behavior.

#### Self-rated health

Self-rated health was measured by the question “How do you rate the quality of your life at present: very good, good, fair, bad, or very bad?” Participants’ level of self-rated health was classified into good (“very good” or “good”), moderate (“fair”), and poor (“bad” or “very bad”) based on their responses.

#### Covariates

Covariates were collected, including age, gender, marital status, education level, work status, and place of residence. Age was classified into three categories (18–44, 45–59, and ≥ 60). Marital status was dichotomized into married and others (never married, divorced, widowed, separated, etc.). Education level was classified into four categories (primary school and below, middle school, high school, and college and above). Work status was dichotomized into employed and unemployed or retired. Place of residence was divided into urban areas and rural areas.

### Statistical analysis

Data analysis was performed using IBM SPSS Statistics Version 22.0 (SPSS, Inc., Chicago, IL). Descriptive statistics including mean and standard deviation (*SD*), frequency and percentage were conducted. The comparison of diet-related knowledge, attitudes, and behaviors in urban and rural residents was conducted using the chi-square test. Ordinal logistic regression analysis was applied to examine the association between diet-related KABs and self-rated health. In all analyses, a *p*-value of < 0.05 was considered statistically significant.

## Results

### General information

Participants’ characteristics are presented in Table 1. A total of 12,814 adult individuals were involved in this study, with an average age of 52.6 years (*SD* = 15.3). Among the participants, 46.9% (*n* = 6016) were male, 86.1% (*n* = 11,038) were married, 32.2% (*n* = 4132) had an education level of primary school and below, 46.0% (*n* = 5891) were currently employed, 60.5% (*n* = 7753) lived in rural areas. 8.5% (*n* = 1089), 40.3% (*n* = 5159), and 51.2% (*n* = 6566) of the participants had poor, moderate, and good self-rated health, respectively. Participants with different demographic characteristics demonstrated significant differences in self-rated health (all *p*-values < 0.05).

**Table 1:** General information

**Variables**	**All participants** (*n* = 12,814)	**Self-rated health**			***p*-value**
		**Poor** (*n* = 1089, 8.5%)	**Moderate** (*n* = 5159, 40.3%)	**Good** (*n* = 6566, 51.2%)	
**Age**					< 0.001
18–44	3887 (30.3)	173 (4.5)	1323 (34.0)	2391 (61.5)	
45–59	4375 (34.1)	307 (7.0)	1776 (40.6)	2292 (52.4)	
≥ 60	4552 (35.5)	609 (13.4)	2060 (45.3)	1883 (41.4)	
**Gender**					0.033
Male	6016 (46.9)	472 (7.8)	2418 (40.2)	3126 (52.0)	
Female	6798 (53.1)	617 (9.1)	2741 (40.3)	3440 (50.6)	
**Marital status**					< 0.001
Married	11,038 (86.1)	868 (7.9)	4474 (40.5)	5696 (51.6)	
Others (never married, divorced, widowed, separated, etc.)	1776 (13.9)	221 (12.4)	685 (38.6)	870 (49.0)	
**Education level**					< 0.001
Primary school and below	4132 (32.2)	585 (14.2)	1917 (46.4)	1630 (39.4)	
Middle school	4068 (31.7)	286 (7.0)	1723 (42.4)	2059 (50.6)	
High school	2845 (22.2)	148 (5.2)	1024 (36.0)	1673 (58.8)	
College and above	1769 (13.8)	70 (4.0)	495 (28.0)	1204 (68.1)	
**Work status**					< 0.001
Employed	5891 (46.0)	810 (11.7)	2965 (42.8)	3148 (45.5)	
Unemployed or retired	6923 (54.0)	279 (4.7)	2194 (37.2)	3418 (58.0)	
**Place of residence**					< 0.001
Urban areas	5061 (39.5)	404 (8.0)	1925 (38.0)	2732 (54.0)	
Rural areas	7753 (60.5)	685 (8.8)	3234 (41.7)	3834 (49.5)	

### Diet-related KABs

Table 2 summarizes the results of diet-related KABs. The proportion of knowing about CFP/DGCR and having adequate dietary knowledge literacy were 27.1 and 34.3%. 24.3% of the participants held positive attitude towards healthy eating. 27.6 and 65.9% of the participants reported the behavior of proactively looking for nutrition knowledge and preferring eating fruits & vegetables.

**Table 2:** Diet-related KABs in urban and rural residents

**Variables**	**All participants** (*n* = 12,814)	**Urban** (*n* = 5061)	**Rural** (*n* = 7753)	***χ***^***2***^	***p*-value**
**Diet-related knowledge**					
Knowing about CFP/DGCR	3468 (27.1)	1955 (38.6)	1513 (19.5)	566.73	< 0.001
Having adequate dietary knowledge literacy	4390 (34.3)	1896 (37.5)	2494 (32.2)	8.12	< 0.001
**Diet-related attitudes**					
Holding positive attitude towards healthy eating	3109 (24.3)	1313 (25.9)	1796 (23.2)	12.86	< 0.001
**Diet-related behaviors**					
Proactively looking for nutrition knowledge	3539 (27.6)	2048 (40.5)	1491 (19.2)	690.72	< 0.001
Preferring eating fruits & vegetables	8444 (65.9)	3484 (68.8)	4960 (64.0)	32.25	< 0.001

CFP: Chinese Food Pagoda; DGCR: Dietary Guidelines for Chinese Residents

Comparative analysis indicated significantly better diet-related KABs in participants lived in urban areas than those in rural areas (all *p*-values < 0.01). Notably, the proportion of knowing about CFP/DGCR (38.6%) and reporting proactively looking for nutrition knowledge (40.5%) in urban areas were almost the double of which in rural areas (19.5 and 19.2%). As shown in Table 2**.**

Besides, we present the detailed results of 17 dietary knowledge questions in Table 3. The proportion of correctly answering to the 17 questions ranged from 28.1% (Q14, *refined grains contain more vitamins and minerals than unrefined grains*) to 83.2% (Q9, *consuming beans and bean products is good for one’s health*), with an average of 64.9%. Only 1.1% (*n* = 143) of the participants made correct answers to all 17 questions. Urban participants reported significantly higher proportion of correctly answering to dietary knowledge questions (except for Q9 and Q14) than rural residents (*p*-value < 0.01).

**Table 3:** The proportion of correctly answering on 17 dietary knowledge questions

**Items**	**Question**	**Total** (*n* = 12,814)		**Urban** (*n* = 5061)		**Rural** (*n* = 7753)		***χ***^***2***^	***p*-value**
		n	%	n	%	n	%		
Q1	Choosing a diet with a lot of fresh fruits and vegetables is good for one’s health.	9715	75.8	4036	79.7	5679	73.2	70.52	< 0.001
Q2^a^	Eating a lot of sugar is good for one’s health.	9325	72.8	3953	78.1	5372	69.3	120.16	< 0.001
Q3	Eating a variety of foods is good for one’s health.	9647	75.3	3989	78.8	5658	73.0	56.13	< 0.001
Q4^a^	Choosing a diet high in fat is good for one’s health.	9239	72.1	3933	77.7	5306	68.4	130.92	< 0.001
Q5	Choosing a diet with a lot of staple foods (rice and rice products, wheat and wheat products) is not good for one’s health.	5283	41.2	2156	42.6	3127	40.3	6.50	0.011
Q6^a^	Consuming a lot of animal products daily (fish, poultry, eggs and lean meat) is good for one’s health.	7965	62.2	3310	65.4	4655	60.0	37.41	< 0.001
Q7	Reducing the amount of fatty meat and animal fat in the diet is good for one’s health.	8894	69.4	3682	72.8	5212	67.2	44.05	< 0.001
Q8	Consuming milk and dairy products is good for one’s health.	10,499	81.9	4241	83.8	6258	80.7	19.63	< 0.001
Q9	Consuming beans and bean products is good for one’s health.	10,658	83.2	4213	83.2	6445	83.1	0.03	0.865
Q10	Physical activities are good for one’s health.	9911	77.3	4006	79.2	5905	76.2	15.63	< 0.001
Q11	Sweaty sports or other intense physical activities are not good for one’s health.	5717	44.6	2400	47.4	3317	42.8	26.66	< 0.001
Q12^a^	The heavier one’s body is, the healthier he or she is.	9767	76.2	4042	79.9	5725	73.8	61.29	< 0.001
Q13	Eating salty foods can cause hypertension.	8888	69.4	3784	74.8	5104	65.8	115.04	< 0.001
Q14^a^	Refined grains (rice and wheat flour) contain more vitamins and minerals than unrefined grains.	3595	28.1	1343	26.5	2252	29.0	9.56	0.002
Q15^a^	Lard is healthier than vegetable oils.	7432	58.0	3131	61.9	4301	55.5	51.33	< 0.001
Q16^a^	Vegetables contain more starch than staple foods (rice or wheat flour).	5700	44.5	2579	51.0	3121	40.3	142.04	< 0.001
Q17	Eggs and milk are the important sources of high-quality protein.	9036	70.5	3704	73.2	5332	68.8	28.69	< 0.001

^a^negative item

### Association between diet-related KABs and self-rated health

Adjusted *ORs* with 95% *CI* were computed to clarify the association between diet-related KABs and self-rated health (Table 4). Participants who knew about CFP/DGCR (*OR* = 1.11, 95% *CI* = 1.08–1.15), had adequate dietary knowledge literacy (*OR* = 1.12, 95% *CI* = 1.10–1.15), held positive attitude towards healthy eating (*OR* = 1.14, 95% *CI* = 1.09–1.19), proactively looked for nutrition knowledge (*OR* = 1.11, 95% *CI* = 1.08–1.15), and preferred eating fruits & vegetables (*OR* = 1.09, 95% *CI* = 1.07–1.12) had significantly better self-rated health.

**Table 4:** Logistic regression analysis predicting the association between diet-related KABs and self-rated health

**Variables**	**Poor**	**Moderate**	**Good**	***OR*(95% *CI*)**^**a**^
**Diet-related knowledge**				
Knowing about CFP/DGCR	176 (16.2)	1122 (21.7)	2170 (33.0)	1.11 (1.08, 1.15)^*^
Having adequate dietary knowledge literacy	256 (23.5)	1514 (29.3)	2620 (39.9)	1.12 (1.10, 1.15)^*^
**Diet-related attitude**				
Holding positive attitude towards healthy eating	941 (86.4)	4761 (92.3)	6180 (94.1)	1.14 (1.09, 1.19)^*^
**Diet-related behaviors**				
Proactively looking for nutrition knowledge	180 (16.5)	1153 (22.3)	2206 (33.6)	1.11 (1.08, 1.15)^*^
Preferring eating fruits & vegetables	649 (59.6)	3174 (61.5)	4621 (70.4)	1.09 (1.07, 1.12)^*^

*DGCR* Dietary Guidelines for Chinese Residents, *OR* odds ratio, *CI* confidence interval

^a^*OR* adjusted for age, gender, marital status, education level, work status, and place of residence

^*^p-value < 0.01

## Discussion

Dietary patterns of Chinese residents have gradually changed in recent years as people’s living standard rises. Unhealthy dietary structure, such as high-energy, high-fat, and high-sugar intake, has been increasingly prominent [19, 20]. Advocating an appropriate diet and promoting diet-related health have becoming a noteworthy topic in China [3]. In this study, we found that rural residents’ diet-related KABs were significantly worse than urban residents. Diet-related KABs of Chinese adult residents were associated with their self-rated health.

This study reported the proportion of 27.1% for knowing about CFP/DGCR and 34.3% for having adequate dietary knowledge literacy, which was consistent with Li et al.’s finding (27.0 and 36.0%) [18]. Compared with the data of former years [21], the proportion of knowing about CFP/DGCR in Chinese adults showed a clear upward trend (2004, 7.8%; 2006, 11.9%; 2009, 14.6%; and 2011, 24.4%), but still at a low level. Only about one-third of the participants had adequate dietary knowledge. And the proportion of correctly answering was extremely low to several dietary knowledge questions. For example, the proportion of correctly answering were 28.1, 41.2, and 44.5% to Q14 (*Refined grains contain more vitamins and minerals than unrefined grains*), Q5 (*Choosing a diet with a lot of staple (rice and rice products, wheat and wheat products) foods is not good for one’s health*), and Q16 (*Vegetables contain more starch than staple foods*). It can be seen that certain diet-related knowledge is still poor in Chinese adult residents and needs to be strengthened in a targeted way.

In this study, 24.3% of the participants held positive attitude towards healthy eating, which was lower than Lê et al.’s results [22] from adults aged 35–64 years in northern and north-eastern France. They reported that 29–43% of the adults’ perceived role of eating was “health”, that is, held positive attitude towards eating. The health belief model (HBM) holds that individual behavior is the external manifestation of psychological activities and the adoption of healthy behaviors is related to individuals’ perceived behavioral benefits and barriers [4]. Previous studies have reported significant association between attitudes towards healthy eating and diet quality, as well as the association between higher diet quality and lower incidence of hypertension and other diseases [22–24]. Therefore, it is necessary to guide and direct Chinese residents to develop healthy diet-related attitudes, so as to lay a good foundation for the adoption of healthy eating behavior.

We found that 27.6% of the Chinese adult residents proactively looked for nutrition knowledge and 65.9% preferred eating fruits & vegetables. In consistent with Li et al.’s report. However, Ouyang et al. [25] and Li et al. [26] mentioned that Chinese residents’ actual intake of fruits and vegetables has not yet met the international recommendation. Zhang et al. [27] even reported a decrease in the consumption of vegetables. These might be due to the increased eating of ultra-processed foods in recent years, which contributed to the decrease of overall diet quality [28, 29]. Thus, behavioral interventions are needed to promote the intake of fruits and vegetables as well as to encourage residents to actively acquire dietary knowledge.

Comparative analyses found significantly worse diet-related KABs in rural areas than in urban areas. Especially for knowing about CFP/DGCR and proactively looking for nutrition knowledge, the proportions in rural people were just about 1/2 of urban residents. Similarly, Gao et al. [30] and He [31] also reported an obvious urban-rural dualistic structure in diet-related KABs. There was thus a need to address the urban-rural gap and to develop interventions targeted rural residents in particular. Although the urbanization in China brings more opportunities to rural residents, there are still not enough health resources available for them. The government should strengthen the effort to promote diet-related health education in rural or poor areas.

The results of regression analysis showed that diet-related knowledge, attitudes, and behaviors were associated with higher self-rated health. Jeruszka-Bielak et al.’s study across [16] five European countries addressed the similar finding. It suggested strong and positive impact of enhancing nutrition-related knowledge and attitudes on health status and quality of life of elderly people. Aune et al. [9] reported that the intake of fruits and vegetables was closely associated with individual health, and could effectively reduce the risks of cardiovascular disease, total cancer, and all-cause mortality. These findings provide important foundation for the implementation of diet-related health education programs.

Chinese government implemented “Appropriate Diet” action as part of the “Healthy China Action (2019-2030)” [3]. Improving diet-related KABs in China is not an easy task. It requires not only the understanding of demand discrepancies between urban and rural residents, but also an insight into the factors preventing Chinese residents from a healthy diet. From the KAB theory perspective, this study reinforced the importance of creating good conditions for diet-related health resources, especially in rural areas. Holistic policy intervention is warranted to target Chinese adults’ overall diet-related knowledge, attitudes, and behaviors rather than relative motivation alone.

Several potential limitations should be mentioned regarding this study. Firstly, limited by the data structure and content of the CHNS database, we only selected two indicators (proactively looking for nutrition knowledge and preferring eating fruits & vegetables) as the measurement of diet-related behaviors. The two indicators may not fully reflect the dietary behaviors of Chinese adult residents. Secondly, all indicators applied in this study were obtained through participants’ self-report, thus are likely to bring measurement errors. For example, there might be a certain deviations between participants’ self-rated health and their actual health status. Due to data limitation, this study is not able to include more objective and comprehensive health status indicators, such as diseases, blood pressure, blood glucose. Thirdly, considering the data timeliness, the 2015 wave of CHNS may not fully reflect China’s current situation. Despite these limitations, the present study systematically described the dietary condition of Chinese adult residents based on large sample data. In addition, this is the first to explore the associations between diet-related knowledge, attitudes, behaviors and individual health from the KAB theory perspective in China, which might be a valuable reference for the implementation of current “Appropriate Diet” actions and further relevant research.

## Conclusions

Chinese adult residents experienced poor diet-related knowledge, attitudes, and behaviors. Rural people reported significantly worse diet-related KABs than urban residents. Better diet-related knowledge, attitudes, and behaviors were significantly associated with the higher self-rated health. The “Appropriate Diet” action implemented in China is in line with the general scientific path of promoting population health through behavioral intervention. It is necessary to develop targeted interventions towards three dietary dimensions: knowledge, attitudes, and behaviors. Moreover, the focus of policy on rural areas to address the urban-rural gap in dietary health might make great sense.

## Data Availability

The datasets generated and/or analysed during the current study are available from the official CHNS website (https://www.cpc.unc.edu/projects/china).

## Abbreviations

CFP: Chinese Food Pagoda;
CHNS: China Health and Nutrition Survey
*CI*: Confidence interval
DALYs: Disability Adjusted Life Years
DGCR: Dietary Guidelines for Chinese Residents
KABs: Knowledge, attitudes, and behaviors
*OR*: Odds ratio
*SD*: Standard deviation
